# CD4 changes among virologically suppressed patients on antiretroviral therapy: a systematic review and meta-analysis

**DOI:** 10.7448/IAS.18.1.20061

**Published:** 2015-08-07

**Authors:** Nathan Ford, Kathryn Stinson, Howard Gale, Edward J Mills, Wendy Stevens, Mercedes P González, Jessica Markby, Andrew Hill

**Affiliations:** 1 Department of HIV/AIDS, World Health Organization, Geneva, Switzerland; 2 Centre for Infectious Disease Epidemiology and Research, University of Cape Town, Cape Town, South Africa; 3 Veteran Affairs Medical Center, Washington, DC, USA; 4 Global Evaluative Sciences, Vancouver, British Columbia, Canada; 5 Department of Molecular Medicine and Haematology, National Health Laboratory Services, University of the Witwatersrand, Johannesburg, South Africa; 6 Department of Essential Medicines and Health Products, World Health Organization, Geneva, Switzerland; 7 Department of Pharmacology and Therapeutics, Liverpool University, Liverpool, United Kingdom

**Keywords:** antiretroviral therapy, CD4, HIV, treatment monitoring, viral load

## Abstract

**Introduction:**

The effectiveness of antiretroviral therapy (ART) is assessed by measuring CD4 cell counts and viral load. Recent studies have questioned the added value of routine CD4 cell count measures in patients who are virologically suppressed.

**Methods:**

We systematically searched three databases and two conference sites up to 31 October 2014 for studies reporting CD4 changes among patients who were on ART and virologically suppressed. No geographic, language or age restrictions were applied.

**Results and discussion:**

We identified 12 published and 1 unpublished study reporting CD4 changes among 20,297 virologically suppressed patients. The pooled proportion of patients who experienced an unexplained, confirmed CD4 decline was 0.4% (95% CI 0.2–0.6%). Results were not influenced by duration of follow-up, age, study design or region of economic development. No studies described clinical adverse events among virologically suppressed patients who experienced CD4 declines.

**Conclusions:**

The findings of this review support reducing or stopping routine CD4 monitoring for patients who are immunologically stable on ART in settings where routine viral load monitoring is provided.

## Introduction

The management of antiretroviral therapy (ART) depends on monitoring both CD4 cell count and viral load. CD4 cell counts inform decisions for initiation of ART, whereas viral load measurement is considered the gold standard for monitoring the effectiveness of ART and detecting early adherence problems in people living with HIV [1]. In high-income settings, the effectiveness of ART is determined using both viral load and CD4 cell count measurements, which are generally carried out at least every six months. In resource-limited settings, there is a concerted effort to increase access to viral load, and most national guidelines recommended either targeted or routine viral load monitoring. These changes reflect the latest World Health Organization (WHO) guidelines recommending viral load as the preferred approach to monitoring treatment efficacy and detecting adherence problems [2]. Most countries are adopting a similar approach to monitoring ART effectiveness using both CD4 cell count and viral load.

Several recent studies have questioned the added value of CD4 cell count monitoring in patients who are virologically suppressed [3–5], and it has been suggested that reducing or eliminating routine CD4 monitoring could save substantial costs [6] and thereby improve the cost-effectiveness of laboratory monitoring in HIV programmes in developing countries [7].

We systematically reviewed the available evidence to determine the extent of CD4 cell count changes among people living with HIV who remain virologically suppressed on ART.

## Methods

This systematic review was conducted in accordance with the reporting standards of the PRISMA guidelines [8].

### Eligibility criteria

Study eligibility was defined according to a predefined study protocol. Randomized trials and prospective and retrospective cohorts were eligible for inclusion if they reported CD4 changes among patients who were on ART and virologically suppressed. Both research cohorts and routine clinic cohorts were eligible for inclusion. No geographic, age or language restrictions were applied.

### Search strategy

Using a broad search strategy, Medline via PubMed, EMBASE and the Cochrane Database of Systematic Reviews were searched from inception to 31 October 2014 for studies reporting CD4 declines among virologically suppressed patients. All conferences of the International AIDS Society and the Conference on Retroviruses and Opportunistic infections were searched from 2012 to identify studies that may have been completed but not yet published in full.

### Data analysis

Data were extracted in duplicate (NF, KS) using a standardized extraction form. The primary outcome was the proportion of unexplained, confirmed (i.e. at least two consecutive measures) CD4 cell count declines <200 cells/mm^3^ among virologically suppressed patients (as defined by the studies). Studies were not excluded if other thresholds for CD4 cell count decline were applied. Secondary outcomes include the proportion of transient declines, non-HIV reasons for CD4 cell count declines and adverse clinical events. Point estimates and corresponding 95% confidence intervals were calculated for the proportion of virologically suppressed patients experiencing an unexplained, confirmed CD4 cell count decline, and data were pooled using random-effects meta-analysis following transformation [9–11]. If the proportion of transient CD4 cell count declines were reported only for a subset of patients, these proportions were applied to the overall sample and potential differences between extrapolated results and results obtained from cohorts for all patients were explored in sensitivity analysis. To explore potential sources of heterogeneity, we used random-effects meta-regression to assess the potential influence of duration of follow-up, study design and level of economic development of the study setting (low- or middle-income country versus high-income country as defined by the World Bank). All analyses were conducted using Stata version 12.0 (StataCorp. LP, College Station, TX, USA).

## Results and discussion

**Figure 1:**
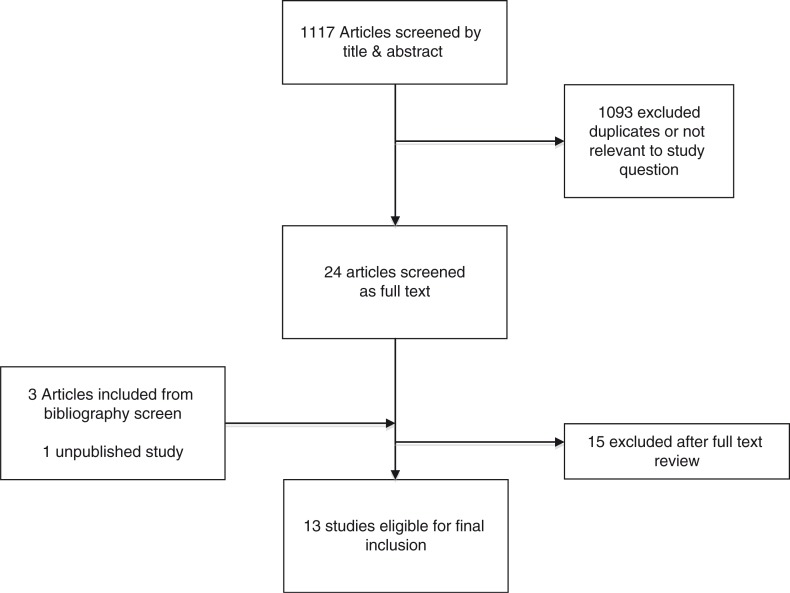
Study selection process.

From a total of 1117 titles screened, 12 published studies [3–5,12–20] were included, representing 20,297 patients. Investigators on one study among adults [20] provided additional data on children (Figure 1). A large initial number of titles were screened because no highly sensitive search strategy could be reliably used to identify the studies of interest. Overall, 13,504 adults and 6793 children on ART were included for review. Most studies were carried out among adults (11 studies) in high-income settings (8 studies); however, around two-thirds of all data (13,776 patients) came from four studies carried out in Africa: South Africa [3,13], Kenya [15] and Uganda [5] (Table 1). The duration of follow-up ranged from 8 to 120 months [3]. Three studies used CD4 criteria other than >200 cells/mm^3^ for study entry and threshold of <200 cells/mm^3^ for decline. One used 500 cells/mm^3^ as the threshold for study entry and 350 cells/mm^3^ as the threshold for study decline [16]; the other two studies used 350 cells/mm^3^ as the threshold for study entry and 200 cells/mm^3^ as the threshold for decline [12,18]. No studies reported data disaggregated by sex.

**Table 1:** Characteristics of included studies

Study [reference]	Setting	Population	Number of subjects	Design	Study period/reporting date*	Study inclusion criteria	Median CD4	Median time on ART	Median follow up
Philips [16]	UK	Adults	166	Hospital clinic, prospective cohort	2001*	Viral load <50 copies/mL and CD4>500 cells/mm^3^	657 cells/mm^3^	23 months	47 weeks
Stephan [17]	Multisite	Adults	230	Randomized trial	2012*	On ART for >60 months. Viral load <20 copies/mL and CD4 >200 cells/mm^3^	571 cells/mm^3^	84 months	48 weeks
Gale [4]	USA	Adults	832	Veterans Affairs Medical Center, retrospective cohort	September 1998 to December 2011	HIV-1 RNA <200 copies/mL and CD4 counts ≥200 cells/mm^3^	NS	NS	92 months
Girard [14]	Multisite	Adults	449	Randomized trial	2013*	CD4 ≥200 cells/µL and HIV-1 RNA<400 copies/mL (taken from results section in abstract)	416	>48 weeks	144 weeks
Whitlock [18]	UK	Adults	141	HIV clinic, retrospective cohort	October 2009 to December 2012	On ART >12 months. CD4 ≥350 cells/mm^3^ at baseline with VL <50 copies/mL	620 cells/mL	NS	30 months
Reynolds [5]	Uganda	Adults	1553	Rakai Health Sciences Program, retrospective cohort	2009–2010	CD4≥200 cells/mm^3^ and HIV VL<400 copies/mL	335	8.3 months	NS
Ford [3]	South Africa	Adults	7250	Primary care clinics, prospective cohort	2001–2012	CD4>200 cells/mm^3^ and HIV VL<400 copies/mL	NS	9–15 months	NS
Kitizo [15]	Kenya	Adults	209	Primary care clinics, retrospective cohort	2011–2012	CD4>200 cells/mm^3^ and HIV VL<1000 copies/mL	257 cells/mL	NS	24 months
Davies [13]	South Africa	Children	5984	Primary care clinics, prospective cohort	2014*	On ART >12 months. VL<400 copies/mL and CD4 ≥25% and 1000 cells/mm^3^ (<5 years) or ≥20% and 500 cells/mm^3^ (≥5 years)	NS	12.9 months	36 months
Chow [12]	Australia	Adults	744	Sexual health centre, retrospective cohort	April 2011 to October 2013	CD4>350 cells/mm^3^ and HIV VL<400 copies/mL	NS	7.3 years	2.5 years
Duncan [19]	UK	Adults	392	Hospital clinic, retrospective cohort	October 2009 to May 2014	CD4>200 cells/mm^3^ and HIV VL<20 copies/mL	567 cells/mL	12 months	3.7 years
Ahn [20]	Multisite, Asia	Adults	1538	HIV clinics, retrospective cohort	September 2003 to March 2013	CD4>200 cells/mm^3^ and HIV VL<400 copies/mL	NS	NS	12 months
Kaen (unpublished)	Multisite, Asia	Children	809	HIV clinics, retrospective cohort	September 2003 to March 2013	NS	NS	NS	12 months

**Figure 2:**
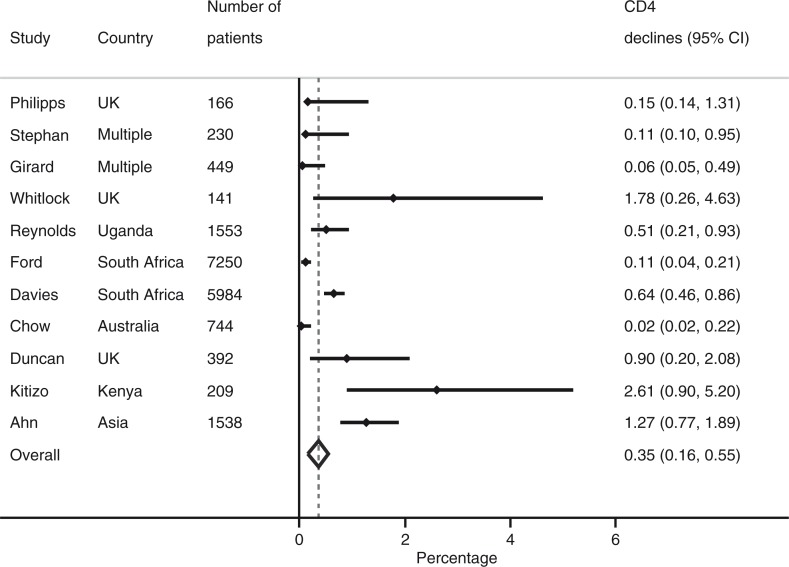
Pooled proportion of virologically suppressed patients experienced an unexplained, confirmed CD4 decline.

Overall, 593 of 20,297 virologically suppressed patients experienced an unexplained, confirmed CD4 decline: the proportion ranged from 0.0% (0.0–0.2%) to 2.6% (95% CI 0.9–5.2%) with an overall pooled proportion of 0.4% (95% CI 0.2–0.6%) (Figure 2). One study was not included in this analysis because CD4 declines were based on a single measure [4]. This estimate did not change if studies that reported transient declines for only a subset of patients were dropped from analysis (0.5%, 95%CI 0.1–0.9%). Results were similar when comparing the study with the shortest duration of follow-up (0.5%, 95%CI 0.2–0.9%) and the longest duration of follow-up (0.1%, 95%CI 0–0.2%). In meta-regression, the results did not appear to be influenced by duration of follow-up, study design or region of economic development. The pooled proportion of transient declines among the total number of CD4 cell count declines (593/750) was 79.5% (95%CI 51.4–100%).

Three studies described non-HIV-related causes of CD4 cell count decline in virologically suppressed patients. In one study, from the USA, 24 of 61 patients who experienced a CD4 cell count decline had documented non-HIV CD4 causes of lymphopenia. There were nine radiation/chemotherapy, seven interferon treatment, three post-surgery, three concomitant severe infection, one viral pneumonia and one steroid treatment [4]. A second study, from the UK, reported 3 of 13 individuals with CD4 cell count decline were on CD4-lowering treatment (interferon or chemotherapy) [18]. The third study, also from the UK, identified six of nine CD4 declines as having a non-HIV-1 cause (two immunosuppressive/corticosteroid therapy, one sepsis, one ART discontinuation, one fulminant liver failure and one vitamin B12 deficiency) [19].

No studies described clinical adverse events among virologically suppressed patients who experienced CD4 declines.

This review identified studies carried out in Asia, Africa, Europe, the USA and Australia and found that CD4 declines among adult and paediatric patients who are virologically suppressed on ART are rare and mainly transient events, or explained by non-HIV factors. This suggests that, for patients stable on ART who are monitored virologically, routine CD4 monitoring could be reduced or stopped.

The debate about using CD4 monitoring for understanding a patient's progression from HIV infection reflects both technological advancements and budgetary restraints. In the early 2000s, affordable viral load monitoring was virtually non-existent in resource-limited settings, but the cost of viral load monitoring has come down considerably in recent years. CD4 monitoring is more accessible but the value of information has decreased in importance: CD4 provides important information about early disease status and early disease improvements when initiating ART but is a crude strategy for informing later disease progress, response to ART or adherence, and provides no inference on whether a patient is likely to transmit to sexual partners.

The latest ART guidelines for South Africa, the country with the largest number of people on ART, recommend stopping routine CD4 monitoring in patients stable on ART, and several other high HIV burden countries are considering a similar change in policy [1]. Several programmes in high-income settings have documented substantial cost savings that could be made if routine CD4 monitoring was stopped [12,15,19]. Following the WHO 2013 recommendation that ART programmes prioritize viral load as the preferred way to monitor ART, countries in resource-limited settings are in the process of scaling up viral load testing capacity; resources spent on CD4 monitoring could be redirected towards supporting viral load [1].

Strengths of this review include the use of a broad search strategy that allowed the identification of studies from programmes in high- and low-income settings, with differing burdens of HIV and comorbidities. Although there is reason to consider the findings to be broadly applicable for adults, more data are needed for children for whom we were only able to identify two studies. The main limitation of this review is the small number of countries contributing data, which may be partly explained by the limited access to laboratory technology to measure CD4 cell count and viral loads in resource-limited settings. Furthermore, only a minority of studies were able to assess and exclude non-HIV causes of CD4 declines, and the actual proportion of HIV-associated CD4 declines in virologically suppressed patients is lower than estimated by our review, and likely explained by biological and analytical variability in the majority of cases. Most studies described CD4 changes over the short term, although the results of these studies were consistent up to 10 years of follow-up. Finally, publication bias is an important concern with any systematic review. Publication bias of implementation sciences is a largely unrecognized important bias, and we cannot rule out this concern.

## Conclusions

CD4 cell counts will continue to be important to support decisions regarding ART initiation, assessing baseline risk of disease progression, and starting and stopping prophylaxis. For individuals on ART, CD4 cell counts will be important in cases where treatment is failing and viral load is detectable. CD4 cell count monitoring will have value among patients taking concomitant immunosuppressive therapy and may also be warranted among individuals with suboptimal CD4 reconstitution. Nevertheless, the findings of this review support recent policy considerations to reduce or eliminate routine CD4 monitoring for patients who are immunologically stable on ART in settings where routine viral load monitoring can be provided.
