# Corrigendum

**DOI:** 10.7448/IAS.18.1.20653

**Published:** 2015-09-02

Regarding the paper ‘CD4 changes among virologically suppressed patients on antiretroviral therapy: a systematic review and meta-analysis’ by Nathan Ford et al.

Published in *Journal of the International AIDS Society*, Citation: Ford N et al. *Journal of the International AIDS Society* 2015, **18**:20061. http://www.jiasociety.org/index.php/jias/article/view/20061 ∣ http://dx.doi.org/10.7448/IAS.18.1.20061

One of the authors names has been incorrectly displayed. Mercedes P González should read *Mercedes Pérez González*.

The correct title and author names are displayed here.

CD4 changes among virologically suppressed patients on antiretroviral therapy: a systematic review and meta-analysis

*Nathan Ford, Kathryn Stinson, Howard Gale, Edward J Mills, Wendy Stevens, Mercedes Pérez González, Jessica Markby, Andrew Hill*

